# Unnecessary and Unkind

**Published:** 1872-05

**Authors:** W. E. Driscoll


### "Unnecessary and Unkind."

BY W. E. DRISCOLL.

In the REGISTER for March is a letter from the Secretary of the Kansas State Dental Society in which he says my judgment of his society, in a communication to the REGISTER of December last, he thinks, is unkind and unnecessary.

In that communication I said: "We would not be understood as desiring to cast discredit upon them because of not having our advantages, etc." Thus disclaiming any unkind motive at the time of writing; and supposed all would so understand it. And I hope the Secretary will give me credit for candor in this statement now that his attention is called to it.

He speaks of the meeting being in November. The report says September in several places. He should be more exact. Carelessness is the cause of most of the misunderstanding and controversy upon all questions.

In my former communication I never so much as thought of criticising the manner in which the Secretary did his task. But now I will risk the declaration, that crowding these reports of society meetings, with matter that is of no possible benefit or interest to the profession, for the purpose of getting in each member's name, is an imposition upon readers and publishers, and a discredit to the profession.

No one should desire to see his name figure in the reports without it is connected with something creditable to him.

The Secretary says my judgment of the Kansas society was unnecessary.

I really left every one to judge for himself, and refrained from all quotations, so that all would compare the reports for themselves.

I used the report of a new society and an old one to show the difference a few years of association would make in the advancement of the profession in modes of practice.

If there is no difference apparent—if those who have here tofore had the advantages of associations have advanced no further than those who have been deprived of them, why go to the expense of organizing societies at all? And when I spoke of our Kansas brethren not having our advantages, it is plain enough that I was speaking of the advantages of past association.

But this writer thinks I did not mean what I said. And if I am "acquainted with the educational facilities that Kansas affords," etc., I "can not mean advantages." Yes I can, the advantages of past association, as I said before.

He says: "It certainly would have been better to give the extracts and comparisons in his paper." I did not deem it necessary, but in deference to his judgment I will give one extract from each report. First from the Kansas report; subject: Alveolar abscess.

"Dr. Patterson, * * * cleaned out the cavity and roots thoroughly, filled the crown cavity and major part of the pulp cavity, leaving the nerve cavity and lower part of the pulp chamber vacant; and after completing, made an opening with a small drill under the edge of the gum into the unfilled portion of the pulp cavity, affording a passage for any matter that would accumulate," etc., etc.

Compare the above with the following from the Lake Erie report: subject; the same, alveolar abscess.

"Dr. McDonneld has been treating abscess for six years and is confident that he has cured ninety-nine in one hundred cases in the superior maxilla. In the inferior maxilla he is not so successful. Injects an escharotic, usually creo sote, through the pulp canal," etc., etc.

Which method would a dentist choose if his own mouth was to be treated?" But it is idle to reprint the two reports. They are already accessible, and speak for themselves, and much fairer than quotations can. To conclude, nothing but an entire misconstruction of the letter and spirit of my communication will give the least ground for this gentleman's complaint. Having spent over ten years "Beyond the Mississippi" I do not regard Kansas as "outside," and shall allow no one to exceed me in rejoicin g at the success of the profession there, now that they have organized for it.

